# How to overcome information and communication barriers in Human Papillomavirus vaccination? A SWOT analysis based on the opinions of European family doctors in contact with young people and their parents

**DOI:** 10.1080/13814788.2024.2393858

**Published:** 2024-08-30

**Authors:** Hüsna Sarıca Çevik, A. Gülsen Ceyhun Peker, Süleyman Görpelioğlu, Shlomo Vinker, Mehmet Ungan

**Affiliations:** a Department of Family Medicine, Faculty of Medicine, Ankara University, Ankara, Turkey; b Türkiye İş Bankası, Ankara, Turkey; c Department of Family Medicine, Faculty of Medicine, Tel Aviv University, Tel Aviv, Israel

**Keywords:** General practice, health communication, Human papillomavirus, primary prevention, vaccines

## Abstract

**Background:**

Family doctors (FDs)/General practitioners (GPs) are the key contact points for young people and their parents regarding Human Papillomavirus (HPV) vaccination. However, their recommendations are influenced by communication skills.

**Objectives:**

Under the EU4Health project, PROTECT-EUROPE, WONCA Europe led a task to identify and analyse strategies for clinicians’ interpersonal communication skills when discussing HPV and its vaccination with young people and their parents.

**Methods:**

Strengths, Weaknesses, Opportunities, Threats (SWOT) analysis using qualitative data focused on HPV vaccine acceptance and communication with the target population. FDs/GPs, members of WONCA Europe, were recruited using convenience and snowball sampling through surveys at conferences and emails.

**Results:**

223 FDs/GPs from 36 countries participated. Strengths included face-to-face communication, extensively used to promote the HPV vaccine. Weaknesses involved financial constraints, limited knowledge about gender-neutral vaccination, safety concerns, and time pressure during the consultations. Opportunities included confidentiality, open dialogue, trusting relationship between FDs/GPs and the target population, continuing medical education, school training, and questions & answers sessions to increase vaccine communication. Threats included social norms and cultures, stigmas against HPV, and anti-vaccination movements hindering discussions on HPV vaccination.

**Conclusion:**

It is crucial to train FDs/GPs to address knowledge gaps, enhance communication skills, and maintain a trusting relationship with patients when discussing HPV vaccination. Overcoming financial barriers and ensuring gender-neutral vaccination programs are accessible across Europe are also essential. Providing accurate information through the web- and school-based channels and developing community-oriented approaches targeting sociocultural factors and different needs to eliminate HPV vaccine stigmas should be considered when recommending the vaccine.

## KEY MESSAGES

Face-to-face, trust-based communication is a strength and opportunity for FDs/GPs to promote the HPV vaccine in all countries.

The lack of free-of-charge, nationally implemented gender-neutral vaccination represents a policy weakness needing improvement.

Stigma and vaccine hesitancy pose major threats, which FDs/GPs must proactively address, supported by robust public health policies.

## Introduction

Human Papillomavirus (HPV) is the most frequent sexually transmitted infection (STI) that can lead to cervical, anal, penile, vaginal, vulvar, and oropharyngeal cancers [1]. 31.1% of cancer cases attributed to infections in both sexes worldwide were caused by HPV in 2020 [2].

The Eurobarometer survey on Europeans’ attitudes towards vaccination (2019) concluded that 79% of respondents would consult a physician for information about vaccines. Physicians and other healthcare professionals (HCPs) were considered the most reliable sources of vaccine information. 2/3 of the participants stated they were vaccinated upon physicians’ recommendation. However, 48% of the participants noted that vaccines can cause serious side effects, and 38% stated that the vaccine can cause the disease it should be protected from [3].

HPV vaccination is associated with a reduced risk of HPV-associated cancers [4]. Effective HPV vaccination programs have critical importance in mitigating both the health and economic impacts of HPV-related cancers. In 2017, an estimated 7,085 HPV-attributable cancer deaths occurred in the United States, resulting in 154,954 years of potential life lost and a present value of future lifetime productivity loss of $4.2 billion. 91% of these deaths and economic burdens were attributable to high-risk HPV types targeted by the 9-valent HPV vaccine [5,6]. However, according to the results of the Vaccine Confidence Project, the importance of the HPV vaccine decreased between 2020 and 2022, which shows that society has reduced confidence in HPV vaccines [7]. Conversely, countries such as Ireland have programs aimed at eliminating cervical cancer through vaccination [8].

Family doctors (FDs)/general practitioners (GPs) are the key contact points and trusted sources of information for young people and their parents/caregivers regarding HPV vaccination. However, HCPs’ recommendations vary between settings and are affected by multi-level factors, which also result in a lack of confidence and knowledge to provide recommendations to the target audience [9].

One of the flagship commitments of Europe’s Beating Cancer Plan is to support Member States in extending routine vaccination against viruses that can cause cancers. PROTECT-EUROPE is an EU4Health project with the participation of WONCA Europe and other European organisations. One of the project’s aims is to optimise one-to-one communication to leverage patients’ trust in clinicians to help increase vaccine uptake [10]. WONCA Europe has led a task to identify and analyse strategies for clinicians’ interpersonal communication skills focused on HPV and its vaccination when talking with young people and their parents through a SWOT analysis workout.

## Methods

### Participants and sampling

In this research, FDs/GPs who are members of WONCA Europe, especially members of the European General Practice Research Network (EGPRN), were reached.

Since the study was mainly based on SWOT analysis, which is a qualitative method, no sample size was calculated. An effort was made to reach as many participants from as many European countries as possible, using convenience and snowball sampling.

### Data collection

Data form included sociodemographic characteristics, the status of HPV vaccine in the National Vaccination Programme, vaccination recommendation and the resources/facilitators FDs/GPs use when recommending the vaccine, the status of physicians vaccinating their children, areas FDs/GPs require a greater understanding of the HPV vaccine and most effective approaches for them to educate about HPV and vaccination. Additionally, five open-ended tables inquired about the benefits and perceived risks of HPV vaccination, and SWOT tables for HPV vaccine acceptance of the target population, recommendations by FDs/GPs, and communication between FDs/GPs, young people and their parents (see Supplementary material).

Before distribution, the survey was piloted with five academic FDs/GPs from different European countries, and the points that needed to be improved were discussed.

The data collection process, which started as hard copy at the Bulgaria GP Research and Education Conference in March 2023, continued through hard copy and online (RedCap platform) at EGPRN Meetings (May and October 2023) and WONCA Europe Conference (June 2023). European Cancer Organisation sent the survey via its networks in July 2023. Additionally, the online survey was emailed to the EGPRN and WONCA Europe members twice in August 2023.

### Analysis

SWOT (Strengths, Weaknesses, Opportunities and Threats) Analysis is an audit tool used in many areas, such as health, and business, that reveals the current state of the organisation in evaluation. It guides determining the positive and negative aspects for guiding and developing the process and taking action, accordingly, evaluating the resources and capabilities related to the operating environment of medical practices, and deciding on future applications in line with the findings. Data collection for SWOT analysis can be done in a meeting/discussion/brainstorming environment, or it can be done through written forms. SWOT are usually presented in a fourfold tabular format to provide convenience to the reader [11,12]. The answers to the open-ended questions were first categorised separately by HSÇ, GCP, SG, and MU, then combined by discussing and presented in the fourfold SWOT tables following the order of frequency of the answers.

IBM SPSS Statistics version 23.0 software (Armonk, NY: IBM Corp) was used for quantitative analyses. Descriptive statistics were reported as mean(±standard deviation) and median (minimum-maximum) regarding variable distribution and as number (n) and percentage (%) for nominal variables. The significance of the difference between HPV vaccine recommendation and sociodemographic data was measured using Mann-Whitney-U (MWU), Fisher’s Exact test, and Pearson Chi-Square, with a significance *p* < 0.05.

### Ethical considerations

Ethical approval (2022/411) was received from the Ankara University Faculty of Medicine Human Research Ethics Committee for this research.

## Results

The survey reached 282 FDs/GPs; 52 incomplete forms were excluded. Answers were received from 36 countries in the WHO Europe region: Albania, Armenia, Austria, Belgium, Bosnia and Herzegovina, Bulgaria, Croatia, Czech Republic, Denmark, Estonia, Finland, France, Georgia, Germany, Greece, Hungary, Ireland, Israel, Italy, Kosovo, Latvia, Lithuania, Luxembourg, Malta, Netherlands, North Macedonia, Norway, Portugal, Romania, Serbia, Slovakia, Spain, Sweden, Turkey, Ukraine and the United Kingdom.

There was also one response each from India, Pakistan, Peru, Morocco, Lebanon, Saudi Arabia and the USA, but these responses were not included due to regional differences, and the study was completed with 223 FDs/GPs.

The mean age of the participants was 43.83 ± 12.08 years (median: 43, min: 26, max: 75). The median length of medical practice as an FD/GP was 14 years (min:0, max:41, IQR:20). 72.6% of the participants were female (*n* = 162), and 82.5% were practising in urban areas (*n* = 184).

92.4% (*n* = 206) of the participants indicated they recommend HPV vaccination. 74.2% (*n* = 153) of them stated they apply face-to-face presentations and education programs when recommending the vaccine. Only 22.3% (*n* = 94) of the participants with children between the ages of 9–26 had had their children vaccinated against HPV.

HPV vaccination recommendations, the presence of HPV vaccination in the national vaccination programme, FDs’/GPs’ information needs regarding HPV vaccination, and the channels they want to receive information are given in Table 1.

**Table 1.** The status of HPV vaccination in the national vaccination programmes, FDs’/GPs’ HPV vaccination recommendations, practices and information needs.

	n	%
**HPV vaccine included in the National Vaccination/Immunisation Programme**		
Yes	169	75.8
No	50	22.4
I do not know	4	1.8
Total	223	100.0
**The groups to whom the vaccine is administered free of charge (*n* = 169)**		
9-14 years old girls	157	92.9
9-14 years old boys	108	63.9
Catch-up vaccination, 14-26 years old females	66	39.1
Catch-up vaccination, 14-26 years old males	44	26
Females over 26 years old	8	4.7
Males over 26 years old	6	3.6
I do not know	5	3.0
Other	15	8.9
**Recommending HPV vaccine**		
Yes	206	92.4
No	17	7.6
Total	223	100.0
**The groups to whom you recommend the HPV vaccine (*n* = 206)**		
9-14 years old girls	173	84
9-14 years old boys	134	65
Catch-up vaccination, 14-26 years old females	145	70.4
Catch-up vaccination, 14-26 years old males	104	50.5
Females over 26 years old	90	43.7
Males over 26 years old	50	24.3
Other	8	3.8
**The resources, facilitators and approaches used while recommending the HPV vaccine (*n* = 206)**		
Printed (written information sources such as brochures, leaflets and posters) and screen information (information on promotion screens in the clinic)	84	40.7
Face-to-face presentations and training programs (such as oral information and explanations, slideshows, Q&A sessions, group discussions, video presentations)	153	74.2
Support *via* the Internet and social media (websites and/or social media groups recommended for young people and/or their families, etc.)	54	26.2
Reminders (such as information provided by telephone, text message, email, mail)	41	19.9
Vaccination campaigns and/or incentives	71	34.4
Other	3	1.45
**Areas FDs/GPs require a greater understanding of the HPV vaccine (*n* = 223)**		
Efficacy	109	48.9
Administration method	21	9.4
Side effects	79	35.4
Vaccination schedule	55	24.7
Gender and age range in which the vaccine is recommended	99	44.4
Price of the vaccine (for countries where the HPV vaccine is not administered free of charge)	64	28.7
Country policies on HPV	75	33.6
Other	9	4
**The most effective approach to finding out information about HPV (*n* = 223)**		
Written sources (books, brochures, etc.)	23	10.3
Congress/conference and peer learning	26	11.7
Web-based offline training	38	17.0
Web-based online training	40	17.9
Web-based evidence-based information on reliable websites	54	24.2
Face-to-face training (one-to-one)	17	7.6
Practical education (hands-on training) in a relevant clinic	23	10.3
Other	2	0.9

Only 51 FDs/GPs (22.8%) could estimate the proportion of the target population who received the vaccine after their recommendation. The median of the estimated ratios was 50% (min: 1%, max: 100%, IQR: 65).

Participants (*n* = 223) rated the possible effect of an education programme on HPV for improving their daily practice between 1 to 10 points, with a mean of 7 ± 2 (median: 8).

There was no statistically significant relationship between HPV vaccine recommendation and age (*p* = 0.866, MWU), length of time in medical practice as an FD/GP (*p* = 0.667, MWU), gender (*p* = 0.648, Pearson Chi-Square) and workplace (*p* = 0.508, Fisher’s Exact).

Table 2 shows the benefits and perceived risks of HPV vaccination according to FDs/GPs.

**Table 2.** Benefits and perceived risks of the HPV vaccination according to FDs/GPs.

**Benefits of the HPV vaccination**	Prevention of HPV related-diseases in both sexes
	Highly effective vaccine
	Reducing the risk of cancer
	Reducing the rates of anogenital warts and recurrent respiratory papillomatosis
	Healthy sexual life
	Economic efficiency of the vaccine
	Safety
**(Perceived) risks of the HPV vaccination**	Local and short-term vaccine side effects (pain, swelling etc.)
	Fever, headache and other flu-like symptoms
	Allergic reactions
	Nausea, vomiting, post-vaccination syncope
	Risky health behaviour (postponing cancer screening, risky sexual behaviour)
	Economic cost to the state
	Other *(Infodemic, increasing opposition, etc.)*

In the question regarding the (perceived) risks of HPV vaccination, the following statements about the need for FDs/GPs to adequately inform patients before HPV vaccination to prevent wrong perceptions that the vaccine is against all STIs and that cervical cancer screening is not needed after vaccination are noteworthy:

***FD195:*** *“Vaccination might induce screening adherence neglect; we must inform patients well.”*

***FD88:*** *“If not explained sufficiently by family physicians, people may perceive that the HPV vaccine is protective against all STIs, resulting in low protection behaviour against infections during sexual intercourse.”*

In addition, when stating cancer prevention, the majority (80%) of participants specified the benefit of HPV vaccination as sex-based, preventing cervical cancer.

Below are the SWOT analysis results regarding the acceptance of the HPV vaccine for participating country populations and, in general in Europe (Table 3), FDs’/GPs’ views on recommending the HPV vaccine (Table 4), and FDs’/GPs’ perspectives on communication about the HPV vaccine between young people (Table 5) and their parents (Table 6). The questions asked were aimed to reveal the SWOT regarding the subjects.

**Table 3.** SWOT table regarding “acceptance” of the HPV vaccine in general in Europe.

**Strengths:** Preventing HPV-related cancer and diseases Public vaccination programs Evidence-based efficacy Trusted primary healthcare workforce Safety, low side effect profile Rising HPV awareness NGO support Good acceptance for girls and increasing gender-neutral HPV vaccination rates	**Weaknesses:** Reimbursement issues (not free-of-charge for all ages and sex) Infodemic and misinformation Low awareness Weak governmental support No clear primary care follow-up strategy Need for more than one dose Lack of communication with patients The long time lapse between vaccination and possibly to measure outcomes Lack of public confidence in vaccination Less likely application of young people to primary care
**Opportunities:** Extension of reimbursement Educational programs/activities about HPV vaccination Availability of the vaccine in pharmacies Volunteering activities HPV testimonies by first-hand experienced patients National Vaccination Programmes Existing long-term relationships of primary healthcare professionals with patients Government support Increased involvement of WHO, UNICEF and NGOs Increased involvement of pharmaceutical companies Peer education	**Threats:** Anti-vaccination movement Fear of side effects Overloaded PHC System Non-existing National Immunisation plan Cultural and religious factors Conservative misbeliefs on HPV vaccines, e.g. encouraging promiscuity Negative effects of the infodemic regarding vaccines during the COVID-19 pandemic

**Table 4.** Participants’ answers to questions about recommending HPV vaccination.

**What can be done to ensure physicians recommend the HPV vaccine to their patients? (S)** Continuing medical education (CME) / Continuous professional development (CPD) Reminders in electronic medical data Easily available evidence-based and reliable information State program Financial motivation A mass campaign about the vaccine Clear guidelines and protocols Free-of-charge vaccination Undergraduate medical education	**What prevents you from recommending the HPV vaccine? (W)** Lack of time Price Lack of knowledge and awareness Lack of communication with patients Lack of a National Vaccination Programme Population prejudice Cultural and religious factors
**What are the opportunities to increase clinician recommendations of the HPV vaccine? (O)** Clinicians are open to further education about HPV vaccinesIncluding gender-neutral HPV vaccination in National Programmes Reducing workload Public campaigns Reimbursement, free access to vaccines Reminders in electronic medical data (electronic health records) Media involvement Trusting relationships with patients (advantage of being a FD/GP) Having functioning NGOs Evidence-based information School-based programmes	**What threats do you perceive when recommending the HPV vaccine? (T)** Misinformed patients Fear of side effects Prejudice Risk of arguing with patients Being blamed for being paid and being a pharma industry-driven doctor Risk of postponing PAP smear and HPV-DNA screenings Neutral governmental policies Misleading people in social media and infodemic

**Table 5.** SWOT table for communication between young people and FDs/GPs from physicians’ perspectives.

**What is the most effective communication method physicians can use to convince young people to accept the HPV vaccine? (S)** Face-to-face conversation Relationship based on mutual trust Social media/official webpages Role models/success stories Group meetings to promote the HPV vaccine in schools and other places where young people frequently use Educational materials Thematic social events/campaigns	**Barriers to strengthening communication between FDs/GPs and young people (W)** Time constraints Young people do not see their FDs/GPs very often Lack of communication skills of FDs/GPs Social media effects and misinformation Cultural/religious characteristics Lack of knowledge of FDs/GPs Use of medical jargon Generation gap Taboo subject Presence of the parents in the practice
**Opportunities and facilitators for strengthening communication between FDs/GPs and young people to increase vaccine acceptance: (O)** Long-lasting close relationship between FDs/GPs and patients Easy-to-reach medical services and vaccination Annual visits Teamwork in primary care Using digital health Active NGOs Vaccination campaigns Posters/printed materials in waiting rooms FDs’/GPs’ HPV vaccine promotion in social media Education in schools Making evidence-based information available on platforms young people use	**Threats that disrupt strong communication between FDs/GPs and young people? (T)** Misinformation and disinformation through social media and influencers (i.e. the thought of taking the vaccine encourages unhealthy sexual practices) Uneducated parents, school teachers, and religious authorities Anti-vaccine movement Thinking of HPV as a taboo subject (Not only the target population, but also some FDs/GPs consider the topic as a taboo subject) The generation gap between physicians and young people Lack of communication skills of pyhsicians Workload of PHC workers

**Table 6.** SWOT table for communication between parents and FDs/GPs from physicians’ perspectives.

**What is the most effective communication method physicians can use to convince parents to accept the HPV vaccine for their children? (S)** Face-to-face conversations Sessions of questions and answers in small groups, and public seminars by FDs/GPs Reminders, e.g. letters, calls, emails, SMS Social media Using flyers from official institutions Recommendation of relevant safe web pages for those who want to have detailed information Providing leaflets/education videos in the waiting room	**Barriers to strengthening communication between FDs/GPs and parents to HPV vaccine acceptance by parents for their children: (W)** Time constraints Fear of side effects HPV vaccine hesitancy Parents’ lack of knowledge about the necessity of gender-neutral vaccination Price of the vaccine Cultural and religious factors Thinking that their children are not at risk Lack of communication skills of physicians Use of medical jargon
**Opportunities and facilitators for strengthening communication between FDs/GPs and parents to increase vaccine acceptance: (O)** Using open, empathetic dialogue Providing enough time for communication with parents, enabling them to get answers to all their questions Involvement of the mass media Visible and effective, free gender-neutral vaccination programmes School vaccination programmes Using routine annual visits State program of motivation and promotion Building good teamwork in PHC Working with NGOs The easy reach of medical service points Well-child clinics Reminders in medical database Good information in dedicated websites Having vaccines available in stores for FD/GP practices	**Threats that disrupt strong communication between FDs/GPs and parents? (T)** Anti-vaccination movements Vaccine hesitancy (Parents’ perceived) lack of reliable data Parents’ fear of side effects Low awareness Negative social media and networks Distrust of physicians Workload of PHC workforce Culture and religion The belief that vaccine encourages unsafe, uncontrolled sex Lack of communication skills Negative image of recent vaccine programs on public (coronavirus-related discussions in media)

## Discussion

The present analysis provided valuable insights into the SWOT associated with HPV vaccine acceptance, and HPV-related communication between FDs/GPs and the target audience.

### Strengths and weaknesses

Strengths and weaknesses are heavily linked to the target population’s relationship with their FDs/GPs. Almost all FDs/GPs stated that “Face-to-face communication” is the most effective method to convince the target population to accept the HPV vaccine. As emphasised in this study, physicians’ ability to reassure young individuals that their information will be kept confidential is essential [13]. An umbrella review of interventions reported that face-to-face presentations, printed information, and incorporating supplementary elements into both approaches effectively boost HPV vaccination intention, which supports the importance of face-to-face communication [14].

As stated in the present study, including the HPV vaccine in national vaccination programmes is important in vaccination decisions [15]. Especially in conservative societies, proposals at the political levels have a facilitator effect.

In the current study, the most prominent weakness in HPV vaccination was financial constraints, which was also stated as an important barrier in the literature [16,17].

Patients’ perceptions and knowledge of HPV represent critical factors influencing vaccination [16], as revealed in the present study. Limited knowledge about the vaccine’s importance before sexual activity, the need for boys to get vaccinated, and lack of access to information and healthcare services arise as weaknesses [18].

Overloaded FDs/GPs complain about a lack of time as a weakness in vaccine communication. Additionally, the technical jargon used by physicians was mentioned as a communication barrier. HPV vaccine recommendations should be made with a simple dialogue that does not contain medical language and conveys the message repetitively [19].

### Opportunities

In the literature, clinicians expressed their need for education on the efficacy and safety of the HPV vaccine [19], consistent with current findings. CME activities for physicians are also reported as an opportunity to increase vaccination and communication. Online training on HPV vaccine communication was considered highly adaptable and acceptable among FDs/GPs [20]. In the current study, FDs/GPs also prioritised web-based methods for accessing information.

Safety concerns about vaccines lead young people and their parents to avoid vaccination [21]. Perceived risk and knowledge of HPV influence parental decisions against vaccinating girls [22]. In this study, FDs/GPs identified insufficient knowledge and awareness among parents and doubts about the vaccine’s reliability as weaknesses. However, school training and Q&A sessions with FDs/GPs were reported as opportunities to overcome this and, accordingly, to increase vaccination and communication. Parents vaccinate their daughters due to physicians’ advice and high vaccine efficacy, while safety concerns and early vaccination age have been reported as reasons for not getting vaccinated [23], which all were reported in the present study. Additionally, physicians’ perception of vaccine safety and efficacy affects HPV vaccination for young people [24].

Most FDs/GPs mentioned open dialogue and an ongoing, trusting relationship with the target population as an opportunity. The primary healthcare workforce is already well trusted and organised for immunisation. HPV vaccine recommendation of the FD/GP, who has known their population for a long time, ensures that the vaccine is seen as important by the parents [25]. Training on HPV vaccine communication provides physicians with effective communication skills about vaccines, taking into consideration the characteristics and needs of the target population [20].

Herd immunity and the desire to protect their children influence parents’ vaccination decisions [26]. Annual visits were seen as an opportunity to recommend vaccinations. Another frequently mentioned opportunity was using social media and web-based platforms to raise awareness, and combat misinformation by providing evidence-based information about HPV vaccination in schools. Clinicians suggested in the literature that public health education, increasing health literacy, effective use of media, targeted messages for parents, and eliminating stigmas against vaccines and STIs as important steps in increasing the vaccination [19], similar to the recommendations from this study.

### Threats

Major threats stem from different social norms and cultures, lacking awareness regarding gender-neutral vaccination, anti-vaccination movements, vaccine hesitancy, the negative image of recent vaccine programs (coronavirus-related discussions in media) and (social) media-mediated misinformation. The most substantial barrier HCPs perceive to gender-neutral HPV vaccination program in Sweden was reported as parents who are sceptical about vaccines [27]. However, organising meetings with individuals who have first-hand experience with the disease can tackle hesitancy among the target population. Effective communication was a fundamental strength, consistent with literature findings [18]. Vaccine hesitancy, which increased with the effects of the infodemic during the COVID-19 has created a higher level of distrust towards vaccines [28,29]. However, open dialogue, tailored to different parent groups’ specific needs and views, represents a notable opportunity to enhance important interactions [27].

As shown in this study, one of the threats to discussing HPV vaccination, is the stigmatisation of STIs [30], opposition to extramarital affairs and sexual health issues. In the British Jewish community, some mothers reject HPV vaccination for their daughters due to religious laws prohibit sexuality until marriage [25]. The belief that the vaccine encourages unhealthy sexual practices was a significant concern of parents to refuse the vaccine. Parents’ judgments about vaccination norms, avoidance of talking about STIs, and negative perceptions about the HPV [31] are among the obstacles that prevent FDs/GPs from discussing HPV vaccination. Therefore, it is recommended to develop community-oriented approaches and training for HPV vaccination.

FDs’/GPs’ attitudes towards not vaccinating their daughters and distrust of the vaccine’s benefits negatively influence their vaccine recommendations [32]. In the current study, 77.7% of those with children between the ages of 9–26 had not been vaccinated their children. However, FDs/GPs also stated that vaccinating their own children creates confidence among parents, and can be used as an opportunity to increase vaccination.

#### Limitations

In addition to the usual limitations of qualitative studies, although the study included participants from most European countries, the unequal distribution of participants may cause a regional bias. Each country has its unique healthcare system, reimbursement policies, and infrastructure, and the perspectives of the included countries may influence the SWOT analysis.

#### Implications for research

Future research should be encouraged to include a more comprehensive representation of European countries to address the identified limitations and provide a comprehensive understanding of HPV vaccination. In addition, concentrating on refining communication strategies to address the challenges of technical language and time constraints could provide valuable insights into improving vaccine uptake. Evaluating the effectiveness of different communication methods, including digital tools, is important.

#### Implications for practice

Developing training programs for FDs/GPs on effective HPV vaccine communication, particularly in addressing patient concerns, can significantly impact patient care. Additionally, developing simple, non-technical communication materials could be helpful.

Utilising web-based platforms for vaccine education and communication can complement face-to-face interactions and provide accessible resources.

#### Implications for policy

This research yielded similar results to previous studies from various countries worldwide, and groups with different ethnic and sociocultural backgrounds. These results suggest the need for recommendations from international collaborations such as WHO, WONCA, medical associations, and governmental levels.

Gender-neutral vaccination programs should be supported for each country. Additionally, policymakers must address vaccine hesitancy driven by social norms and misinformation through public health campaigns.

## Conclusion

The analysis underscores that face-to-face communication, trust in HCPs, and effective education are key strengths in promoting HPV vaccine acceptance. Supporting FDs/GPs with training to respond to their knowledge gaps, enhance communication skills, and maintain a confidential, trusting relationship with patients is crucial. Financial constraints, communication barriers, and social stigmas present significant challenges. Therefore, it is important to overcome financial barriers to HPV vaccination, make gender-neutral vaccination programmes accessible across Europe, and consider sociocultural differences, when recommending the HPV vaccine. Providing accurate information to the population with alternative channels such as web- and school-based, and developing community-oriented approaches and training to eliminate stigmas against vaccines and STIs, through targeted research, improved practice, and supportive policies will be crucial for enhancing HPV vaccination rates.

## Supplementary Material

Supplemental Material

Supplemental Material
