# Supplementary material for: How to overcome information and communication barriers in Human Papillomavirus vaccination? A SWOT analysis based on the opinions of European family doctors in contact with young people and their parents
Source: Eur J Gen Pract. 2024 Aug 30;30(1):2393858. doi: 10.1080/13814788.2024.2393858 (PMC11370665; doi:10.1080/13814788.2024.2393858)
Supplement: Supplemental Material [file IGEN_A_2393858_SM2521.docx]

**QUESTIONNAIRE FORM**

**1) Age in years:** ………

**2) Gender:** ( ) Female ( ) Male ( ) I do not want to specify

**3) Length of time in medical practice as a family physician/general practitioner (in years):** ……… years

**4) The country currently employed:** ………

**5) Your main workplace:** ( ) Urban ( ) Rural

**6) If you have a child 9 years and older, has he/she received the HPV vaccine?**

( ) Yes ( ) No: (reason) ……

**7) Is the HPV vaccine included in the National Vaccination/Immunization Programme in the country in which you currently work?**

( ) Yes ( ) No ( ) I do not know

**---If your answer is ‘No’ or ‘I do not know’, please proceed to Question 9.**

**--- If your answer is 'Yes', please identify the groups to whom the vaccine is administered free of charge. (Please tick all appropriate options)**

( ) 9-14 years old girls

( ) 9-14 years old boys

( ) Catch-up vaccination, 14-26 years old females

( ) Catch-up vaccination, 14-26 years old males

( ) Females over 26 years old

( ) Males over 26 years old

( ) I do not know

( ) Other (please specify): …..

**8) Do you recommend the HPV vaccine in a professional capacity?**

( ) Yes

( ) No

--- **If your answer is 'No', please state the reasons in your own words:**

……………………………….

--- **If your answer is 'Yes', please indicate the groups to whom you recommend the HPV vaccine. (Tick all appropriate options)**

( ) 9-14 years old girls

( ) 9-14 years old boys

( ) Catch-up vaccination, 14-26 years old females

( ) Catch-up vaccination, 14-26 years old males

( ) Females over 26 years old

( ) Males over 26 years old

( ) Other (please specify): …..

**--- If your answer is 'Yes', please indicate the resources, facilitators and approaches you use when recommending the HPV vaccine? (Check all the options you use in your practice.)**

( ) Printed (written information sources such as brochures, leaflets and posters) and screen information (information on promotion screens in the clinic)

( ) Face-to-face presentations and training programs (such as oral information and explanations, slideshows, Q&A sessions, group discussions, video presentations)

( ) Support via the internet and social media (websites and/or social media groups recommended for the young people and/or their families, etc.)

( ) Reminders (such as information provided by telephone, text message, email, mail)

( ) Vaccination campaigns and/or incentives

( ) Other: ..................

**9) Please estimate the proportion of young people/parents/guardians to whom you recommend the HPV vaccine go on to receive the vaccine? (If you do not know, please leave it blank):** %.……………………………….

**10) Please indicate the areas you require greater understanding of the HPV vaccine (Please tick all appropriate options):**

( ) Efficacy

( ) Administration method

( ) Side effects

( ) Vaccination schedule

( ) Gender and age range in which the vaccine is recommended

( ) Price of the vaccine (for countries where the HPV vaccine is not administered free of charge)

( ) Country policies on HPV

( ) Other: ………………

**11) What do you think are the benefits and risks of the HPV vaccination?**

| **Benefits** |  |
| --- | --- |
| **Risks** |  |

**12) Please rate the possible effect of an education programme on HPV for improving your daily practice (1 point=lowest and 10 points=highest): ...........**

**13) Please indicate the most effective approach you would prefer to find out information about HPV and HPV immunisations? (Please tick only the most effective, one answer)**

( ) Written sources (books, brochures, etc.)

( ) Congress/conference and peer learning

( ) Web-based offline training

( ) Web-based online training

( ) Web-based evidence-based information on reliable websites

( ) Face-to-face training (one-to-one)

( ) Practical education (hands-on training) in a relevant clinic

( ) Other (please indicate): ………………

**Below you will see questions about the clinician and the target population (youth and parents/caregivers) for whom the HPV vaccine is recommended. Write your own opinions on these questions for all three groups (clinician, youth, parents/caregivers) from a family doctor's/general practitioner’s perspective.**

***When answering the questions below, please consider gender-neutral vaccinations, not only for girls and women but also boys and men.***

1. **Please write down any strengths, weaknesses, opportunities, and threats you think of regarding the acceptance of the HPV vaccine for your population in the table below:**

| **Strengths:** | **Weaknesses:** |
| --- | --- |
| **Opportunities:** | **Threats:** |

1. **Questions for GPs/FDs:**

| **What can be done to ensure doctors recommend the HPV vaccine to their patients?** | **What prevents you from recommending the HPV vaccine?** |
| --- | --- |
| **What are the opportunities to increase recommendations of the HPV vaccine by clinicians?** | **What threats do you perceive when recommending the HPV vaccine?** |

1. **Questions about young people from the perspective of FDs/GPs:**

| **What is the most effective communication method doctors can use to convince young people to accept the HPV vaccine?** | **Barriers to strengthening communication between FDs/GPs and young people:** |
| --- | --- |
| **Opportunities and facilitators for strengthening communication between GPs and young people to increase vaccine acceptance:** | **Threats that disrupt strong communication between GPs and young people?** |

1. **Questions about parents from the perspective of GPs/FDs:**

| **What is the most effective communication method doctors can use to convince parents to accept the HPV vaccine for their children?** | **Barriers to strengthening communication between FDs/GPs and parents to HPV vaccine acceptance by parents for their children:** |
| --- | --- |
| **Opportunities and facilitators for strengthening communication between FDs/GPs and parents to increase vaccine acceptance:** | **Threats that disrupt strong communication between FDs/GPs parents?** |

1. **Any additional suggestions:**

**………………………………………………………………………..**
